# Evaluation of Kallistatin Levels in COPD Exacerbations

**DOI:** 10.1155/carj/4440479

**Published:** 2025-06-12

**Authors:** Kadir Burak Akgün, Serdar Doğan, Nursel Dikmen

**Affiliations:** ^1^Department of Pulmonology, Faculty of Medicine, Hatay Mustafa Kemal University, Antakya, Hatay, Türkiye; ^2^Department of Medical Biochemistry, Faculty of Medicine, Hatay Mustafa Kemal University, Antakya, Hatay, Türkiye; ^3^Department of Pulmonology, Gaziosmanpaşa Hospital, Istanbul Yeni Yüzyıl University, Istanbul, Türkiye

**Keywords:** chronic obstructive pulmonary disease, kallistatin, oxidative stress

## Abstract

**Introduction:** Kallistatin is an enzyme with antioxidative and anti-inflammatory properties and has been shown to provide protection against pneumosepsis, acute respiratory distress syndrome (ARDS), and lung fibrosis. This study revealed the use of kallistatin in the clinical management of COPD.

**Materials and Methods:** Forty-eight COPD patients were evaluated during both exacerbation and stable periods. A control group of 30 healthy individuals was also included. In addition to kallistatin, serum levels of TAS, TOS, OSI, VEGF, and TNF-*α* were measured. Data were statistically analyzed for the exacerbation and stable periods of COPD patients, as well as the control group. Correlation analysis of serum parameters was conducted, and regression analysis was performed on those with significant results.

**Results:** Serum kallistatin levels were significantly lower in COPD patients compared to the normal population (*p* < 0.001). Additionally, kallistatin levels were lower during COPD exacerbations compared to the stable period (*p* < 0.001). Kallistatin levels measured during exacerbations were positively correlated with OSI and VEGF (*r* = 0.333, *p*=0.021; *r* = 0.301, *p*=0.037, respectively). The relationship between kallistatin and OSI was strongly supported by regression analysis (*p*=0.049, CI 16.889).

**Conclusion:** Kallistatin is a promising biomarker for distinguishing COPD patients from the normal population and for identifying disease exacerbations.

## 1. Introduction

COPD is a persistent and generally progressive obstructive disease that leads to chronic respiratory complaints and develops due to pathologies in the airways and/or alveoli. Smoking, biomass exposure, and air pollution are the main risk factors for COPD development, but genetic predisposition is also thought to play a role. COPD is diagnosed when the FEV1/FVC ratio is shown to be below 70% in postbronchodilator spirometry. COPD is one of the leading causes of mortality and morbidity worldwide [1].

COPD exacerbation is defined as an increase in dyspnea, cough, and/or sputum production within the last 14 days. These exacerbations have significant negative effects on quality of life, hospital admissions, and disease progression. COPD exacerbation is usually associated with systemic and local increased inflammation, which causes irritation in the respiratory tract due to factors such as infection or air pollution [1, 2].

In COPD, inflammation is observed in the lungs and is thought to be a natural inflammatory response to chronic irritants. The number of macrophages increases in the airways, parenchyma, and vascular structures, along with an increase in activated neutrophils and lymphocytes. These cells activate the inflammatory cascade together with epithelial and structural cells. In addition to the lungs, systemic inflammation is also present in COPD [1, 3].

Oxidative stress is also known to contribute to COPD. Studies have shown that oxidative stress markers are elevated in the respiratory tract and systemic circulation in COPD. Moreover, oxidative stress increases more significantly during COPD exacerbations [1, 4].

Kallistatin, a serine protease inhibitor encoded by the SERPINA4 gene [5], functions as a tissue kallikrein inhibitor and exerts antioxidant, anti-inflammatory, antiangiogenic, antiapoptotic, and antitumor effects through its active site and heparin-binding domain [6, 7]. It is primarily synthesized in the liver and is also widely distributed in the kidneys, eyes, heart, and vascular structures [8]. Its protective role against pulmonary diseases such as pneumonia, pulmonary fibrosis, and ARDS has been well-documented. Although kallistatin has been widely researched, its role in COPD remains extremely limited. This study aims to investigate the relationship between kallistatin levels and COPD, focusing on its potential as a biomarker for disease activity and progression.

## 2. Materials and Methods

### 2.1. Study Participants

Approval for this study was obtained from the Hatay Mustafa Kemal University Clinical Research Ethics Committee on June 6, 2022, under decision number 03. Patients included in the study were diagnosed with COPD according to the GOLD guideline criteria. In accordance with the GOLD guideline, patients with COPD exacerbations were defined as those exhibiting a worsening of dyspnea, cough, and/or sputum production within a 14-day period. Stable patients were defined as those whose respiratory status returned to their baseline condition after the resolution of the exacerbation. Exclusion criteria included being under 18 years old, pregnancy, and additional chronic lung diseases such as asthma or interstitial lung disease, as well as diabetes mellitus, chronic renal failure, rheumatological or oncological diseases, and the use of immunosuppressive drugs. To create a control group, serum blood samples were taken from individuals over 18 years old without known comorbidities, such as diabetes mellitus, chronic renal failure, hypertension, cardiovascular diseases, rheumatological or oncological diseases, or any other significant medical condition. All participants were required to read and sign the informed consent form.

### 2.2. Study Design

Each of the patients included in the study was evaluated during both the exacerbation period and the subsequent stable period. Data collected included patients' age, gender, smoking history, comorbidities, the presence of pneumonia at the onset of exacerbation, use of steroids in treatment, and whether the treatment was outpatient or inpatient. All patients underwent routine lung imaging, and pneumonia was diagnosed by chest radiography and/or thoracic tomography. Patients with evident bronchospasm were considered for steroid intervention; 40 mg of methylprednisolone was administered for 5 days as standard treatment for those with respiratory failure accompanied by wheezing or rhonchi. Additionally, GOLD group classifications, use of home oxygen concentrators, and noninvasive mechanical ventilator usage were recorded. Patients were also grouped based on the severity of exacerbation, which was determined according to the GOLD guidelines (mild: requiring only short-acting bronchodilators; moderate: requiring additional antibiotics and/or steroids; severe: requiring hospitalization).

Within the scope of the study, serum kallistatin, total antioxidant status (TAS), total oxidant status (TOS), oxidative stress index (OSI), vascular endothelial growth factor (VEGF), and tumor necrosis factor-alpha (TNF-*α*) levels were measured in COPD patients during exacerbation and stable periods. COPD exacerbation was defined according to the GOLD guideline criteria. The stable period was defined as a phase in which the patient had received treatment for at least 1 week and exhibited clinical improvement, characterized by the resolution or marked reduction of dyspnea, cough, and/or sputum production.

### 2.3. Collection and Preparation of Samples

Blood was collected from both the control and patient groups into yellow-capped gel biochemistry tubes. The samples were allowed to clot for 20 min before being centrifuged at 1500 × g for 10 min. The supernatants were then transferred to Eppendorf tubes and stored at −80°C until the day of the study for biochemical analysis. Plasma samples were separated by centrifugation and stored at −80°C, a widely accepted method to preserve biomolecules, such as proteins and enzymes, which are sensitive to degradation. This storage condition ensures the integrity of the samples over time, preventing molecular alterations until the day of biochemical analysis.

### 2.4. Parameters Measured by ELISA Method

Serum samples were collected in gel-coated biochemistry tubes and analyzed using the sandwich ELISA method at the ELISA Laboratory of the Faculty of Medicine, Hatay Mustafa Kemal University. The Thermo Scientific MultiscanGo UV (USA) device was used for measuring the ELISA parameters, and the Thermo Scientific washer (Finland) was used for washing during the protocols.

### 2.5. Measurement of Serum Kallistatin (SERPINA4)

Serum kallistatin (SERPINA4) levels were measured using a commercial kit (FineTest, Human SERPINA4 (Kallistatin) ELISA Kit, Cat no: EH0209) with the sandwich ELISA method. Optical density (OD) was read at 450 nm using a microplate reader. Concentrations were calculated using a 4-parameter logistic (4PL) calibration curve. Results are expressed as pg/mL. The assay range is 15.625–1000 ng/mL with a sensitivity of 9.375 ng/mL. Precision is intraassay CV < 8% and interassay CV < 10%.

### 2.6. Serum TAS Measurement

Serum TAS was measured using the colorimetric method with an Elabscience kit (Cat no: E-BC-K801-M) at the Hatay Mustafa Kemal University Medical Biochemistry Laboratory. The analysis range is 0.23–2 mmol Trolox equivalent/L. Precision is intraassay CV < 2.3% and interassay CV < 3.5%. Results are expressed as mmol Trolox equivalent/L.

### 2.7. Serum TOS Measurement

Serum TOS was measured using the colorimetric method with an Elabscience kit (Cat no: E-BC-K802-M) at the Hatay Mustafa Kemal University Medical Biochemistry Laboratory. The analysis range is 2.5–100 μmol H_2_O_2_ equivalent/L. Precision is intraassay CV < 2.3% and interassay CV < 3.5%. Results are expressed as μmol H_2_O_2_ equivalent/L.

### 2.8. Measurement of Serum OSI

The OSI was calculated from the TOS and TAS values. Results are expressed in arbitrary units (AU). Calculation is OSI (AU) = (TOS/TAS) × 100.

### 2.9. Measurement of Serum VEGF

Serum VEGF was measured using a commercial kit (FineTest, Human VEGF ELISA Kit, Cat no: EH0327) with the sandwich ELISA method. The OD was read at 450 nm using a microplate reader. Concentrations were calculated using a 4PL calibration curve. Results are expressed as pg/mL. The analysis range is 31.25–2000 pg/mL with a sensitivity of 18.75 pg/mL. Precision is intraassay CV < 8% and interassay CV < 10%.

### 2.10. Measurement of Serum TNF-*α*

Serum TNF-*α* was measured using a commercial kit (FineTest, Human TNF-*α* ELISA Kit, Cat no: EH0302) with the sandwich ELISA method. The OD was read at 450 nm using a microplate reader. Concentrations were calculated using a 4PL calibration curve. Results are expressed as ng/mL. The analysis range is 15.625–1000 pg/mL with a sensitivity of 9.375 pg/mL. Precision is intraassay CV < 8% and interassay CV < 10%.

### 2.11. Statistical Analysis

Age, gender, and smoking habits between the groups were evaluated using the chi-square test. The normal distribution of the data was evaluated using the Kolmogorov–Smirnov test. Differences in serum parameters between the patient and control groups were analyzed using the independent *t*-test for normally distributed data and the Mann–Whitney *U* test for non-normally distributed data. The Wilcoxon test was used to assess differences between exacerbation and stable period data within the patient group. The Kruskal–Wallis test and Mann–Whitney *U* test were employed to analyze relationships between patient subgroups and data. For correlation analysis, the Pearson test was used for normally distributed data, and the Spearman test was applied for non-normally distributed data. Data found to be correlated with kallistatin were further examined using regression analysis, with age, gender, and pneumonia status as covariates. Additionally, ROC analysis was performed by comparing exacerbation and stable period kallistatin values in patients with the control group's kallistatin values. Statistical significance was defined as *p* < 0.05. All statistical analyses were conducted using the Statistical Package for the Social Sciences (SPSS Inc., Chicago, IL, USA) version 25.

## 3. Results

A total of 48 COPD patients were included in the study. The mean age of the patients was 67.22 years (SD ± 10.48), with a male-to-female ratio of 8.6:1. According to the GOLD classification, 64.6% of the patients were classified in Group E (*n* = 31), and 22.9% of patients had never smoked. The use of home oxygen concentrators and noninvasive mechanical ventilators was 6.3% and 2.1%, respectively (Tables 1 and 2).

Statistically significant differences were found in kallistatin, TAS, TOS, OSI, VEGF, and TNF-*α* values between exacerbation and stable periods (*p* < 0.001 for all comparisons). Additionally, kallistatin, TAS, TOS, OSI, and TNF-*α* levels were significantly different in COPD patients compared to the control group during both exacerbation and stable periods (*p* < 0.001 for each parameter). VEGF levels did not show a significant difference between COPD patients and healthy controls during either exacerbation (*p*=0.72) or stable periods (*p*=0.902) (Table 3).

No significant relationships were observed between cigarette consumption, GOLD groups, steroid intervention, and treatment site. However, stable-period TOS values were significantly higher in patients with pneumonia compared to those without (*p*=0.03). No other significant relationships were identified between pneumonia and the remaining data.

**Figure 1:**
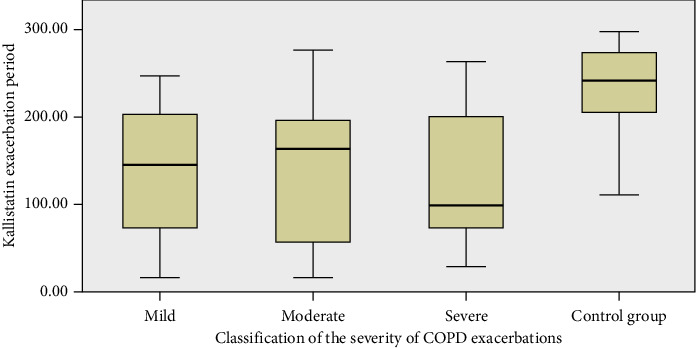
Relationship between kallistatin and COPD severity.

No significant differences were observed between the exacerbation severity groups when analyzing kallistatin levels. However, significant differences were found when comparing the exacerbation groups to the control group (*p*=0.005 for mild vs. control, *p* < 0.001 for moderate vs. control, and *p*=0.004 for severe vs. control) (Figure 1).

**Figure 2:**
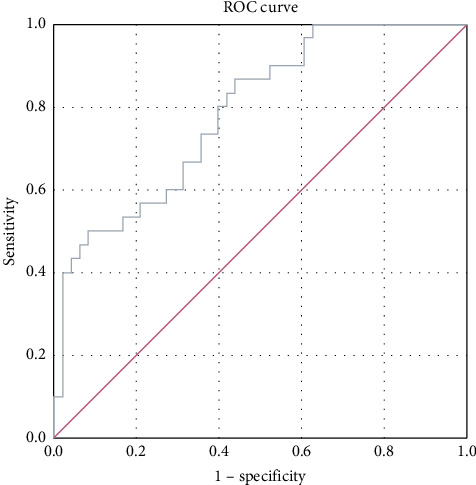
ROC curve for kallistatin during stable COPD period vs. control group.

**Figure 3:**
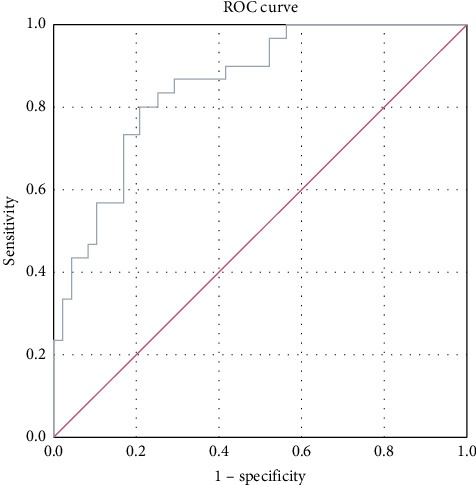
ROC curve for kallistatin during exacerbation COPD period vs. control group.

ROC analysis was performed to compare kallistatin levels in stable COPD patients with those in the normal population. The area under the curve (AUC) for stable-period kallistatin levels was 0.788 (95% CI: 0.688–0.888, *p* < 0.001) (Figure 2). The AUC for exacerbation-period kallistatin levels was 0.853 (95% CI: 0.771–0.936, *p* < 0.001) (Figure 3).

Correlation analysis between kallistatin and other parameters was conducted for both exacerbation and stable periods (Tables 4 and 5). A significant relationship was found between exacerbation-period kallistatin and OSI values (*r* = 0.333, *p*=0.021). Regression analysis confirmed this association (*p*=0.049, CI 16.889). However, no significant predictive relationship was found between exacerbation-period kallistatin and VEGF values (*p*=0.060, CI 1.191) (Table 6).

## 4. Discussion

In our prospective, controlled cohort, serum kallistatin levels were significantly lower in COPD patients than in healthy controls and declined further during exacerbations. This finding parallels observations by Lin et al. [9], who reported lower plasma kallistatin in septic shock versus sepsis and linked Day 1 levels to mortality, as well as Kim et al. [10] and Lin et al. [11], who found reduced kallistatin associated with increased mortality, septic shock, and ARDS in community-acquired pneumonia cohorts. Kallistatin's protective role in pulmonary inflammation is further supported by Huang et al. [12], who demonstrated its efficacy against bleomycin-induced pulmonary fibrosis.

To our knowledge, only Ngo et al. [13] have investigated kallistatin in COPD, associating it with FEV_1_ and FEV_1_/FVC across six heterogeneous cohorts but limited by prebronchodilator measures and assay variability.

We also confirmed that oxidative stress markers (TAS, TOS, and OSI) and inflammatory mediators (TNF-*α* and VEGF) increased during exacerbations, consistent with established COPD pathogenesis [14–18]. The strong positive correlation between kallistatin and OSI suggests that kallistatin may reflect antioxidant capacity and dynamically track oxidative burden in COPD.

Importantly, ROC analysis demonstrated that plasma kallistatin discriminates stable COPD from healthy controls with an AUC of 0.788 (95% CI, 0.688–0.888; *p* < 0.001) and exacerbations from health with an AUC of 0.853 (95% CI, 0.771–0.936; *p* < 0.001), indicating fair-to-good diagnostic accuracy and potential clinical utility pending multicenter validation.

Strengths and Limitations of the Study: This study offers novel insights into kallistatin levels in COPD and their association with exacerbations. Its prospective design with a control group strengthens the validity of the findings. However, the single-center design and small sample size limit the generalizability and statistical power of the results.

## 5. Conclusion

This study provides evidence that kallistatin levels are significantly lower in COPD patients and further decrease during exacerbations. The findings support the potential of kallistatin as a biomarker for COPD exacerbations, suggesting its primary role may be linked to antioxidant mechanisms. However, while kallistatin is known for its antioxidant effects, the observed correlation with oxidative stress markers raises the possibility of its involvement in oxidative processes. Further investigation is needed to explore this potential role and other pathophysiological factors.

## Figures and Tables

**Table 1:** Participant characteristics.

Groups	Age mean ± SD	Sex *n* (%)	Smoking status *n* (%)
COPD (*n* = 48)	67.22 ± 10.48	Male = 43 (89.6) Female = 5 (10.4)	Never smoked = 11 (22.9)
			Formerly smoker = 18 (37.5)
			Active smoker = 19 (39.6)

Control (*n* = 30)	60.40 ± 7.07	Male = 28 (93.3) Female = 2 (6.7)	Never smoked = 9 (30)
			Formerly smoker = 13 (43.3)
			Active smoker = 8 (26.7)

*p*	0.107	0.573	0.494

*Note:p* value was obtained from chi-square test.

**Table 2:** Patient characteristics.

COPD patients (*n* = 48)	*n* (%)
GOLD group	
A	9 (18.8)
B	8 (16.7)
E	31 (64.5)
COPD exacerbation severity	
Mild	10 (20.8)
Moderate	27 (56.3)
Severe	11 (22.9)
Home oxygen concentrator	
Yes	3 (6.3)
No	45 (93.7)
Home NIMV	
Yes	1 (2.1)
No	47 (97.9)
Place of treatment	
Outpatient	37 (77.1)
Inpatient	11 (22.9)
Pneumonia during exacerbation	
Yes	21 (43.8)
No	27 (56.2)
Steroid intervention during exacerbation	
Yes	27 (56.2)
No	21 (43.8)

Abbreviation: NIMV = noninvasive mechanic ventilation.

**Table 3:** Comparison of serum parameters in COPD patients and control group.

Parameters	Exacerbation period (*n* = 48) (group I) mean ± SD	Stable period (*n* = 48) (group II) mean ± SD	Control group (*n* = 30) (group III) mean ± SD	*p*		
				I vs II	I vs III	II vs III
Kallistatin (pg/mL)	135.62 ± 78.14	153.09 ± 80.94	232.26 ± 51.28	<0.001^**a**^	<0.001^**b**^	<0.001^**c**^
TAS (mmol trolox equiv/L)	1.08 ± 0.37	1.43 ± 0.39	1.84 ± 0.08	<0.001^**a**^	<0.001^**b**^	<0.001^**c**^
TOS (μmol H_2_O_2_ equiv/L)	35.44 ± 10.00	22.56 ± 6.89	16.42 ± 4.44	<0.001^**a**^	<0.001^**c**^	<0.001^**c**^
OSI (AU)	3.58 ± 1.40	1.70 ± 0.73	0.89 ± 0.25	<0.001^**a**^	<0.001^**b**^	<0.001^**c**^
VEGF (pg/mL)	535.47 ± 395.84	404.11 ± 315.91	361.17 ± 178.88	<0.001^**a**^	0.072^c^	0.902^c^
TNF-*α* (pg/mL)	238.67 ± 85.09	199.62 ± 77.14	151.26 ± 24.69	<0.001^**a**^	<0.001^**c**^	<0.001^**c**^

*Note:* Values corresponding to the significance level *p* < 0.05 are marked in bold.

Abbreviations: OSI = oxidative stress index, TAS = total antioxidant status, TNF-*α* = tumor necrosis factor-alpha, TOS = total oxidant status, VEGF = vascular endothelial growth factor.

^a^ *p* value obtained from Wilcoxon test.

^b^ *p* value obtained from independent *t*-test.

^c^ *p* value obtained from Mann–Whitney *U* test.

**Table 4:** Correlation analysis of kallistatin and other parameters in exacerbation period.

		Kallistatin exacerbation period	TAS exacerbation period	TOS exacerbation period	OSI exacerbation period	VEGF exacerbation period	TNF-*α* exacerbation period
Kallistatin exacerbation period	*R*	1	−0.108^∗^	0.266	**0.333** ^**∗**^	**0.301**	0.098
	*p*		0.465^∗^	0.067	**0.021** ^**∗**^	**0.037**	0.508

TAS exacerbation period	*R*	−0.108^∗^	1	0.248	**0.674** ^**∗**^	0.242	0.201
	*p*	0.465^∗^		0.089	**0.000** ^**∗**^	0.097	0.171

TOS exacerbation period	*R*	0.266	0.248	1	**0.458**	0.007	−0.418
	*p*	0.067	0.089		**0.001**	0.965	0.316

OSI exacerbation period	*R*	**0.333** ^**∗**^	**−0.674** ^**∗**^	**0.458**	1	−0.213	−0.281
	*p*	**0.021** ^**∗**^	**0.000** ^**∗**^	**0.001**		0.147	0.053

VEGF exacerbation period	*R*	**0.301**	0.242	0.007	−0.213	1	0.054
	*p*	**0.037**	0.097	0.965	0.147		0.714

TNF-*α* exacerbation period	*R*	0.098	0.201	−0.418	−0.281	0.054	1
	*p*	0.508	0.171	0.316	0.053	0.714	

*Note:* Values corresponding to the significance level *p* < 0.05 are marked in bold.

Abbreviations: OSI = oxidative stress index, TAS = total antioxidant status, TNF-*α* = tumor necrosis factor-alpha, TOS = total oxidant status, VEGF = vascular endothelial growth factor.

^∗^ *p* value was obtained from Pearson test. Others were obtained by Spearman test.

**Table 5:** Correlation analysis of kallistatin and other parameters in stable period.

		Kallistatin stable period	TAS stable period	TOS stable period	OSI stable period	VEGF stable period	TNF-*α* stable period
Kallistatin stable period	*R*	1	−0.218	−0.124	0.071	0.265	0.034
	*p*		0.137	0.401	0.633	0.068	0.817

TAS stable period	*R*	−0.218	1	0.204	**−0.543**	0.191	−0.206
	*p*	0.137		0.164	**0.000**	0.193	0.160

TOS stable period	*R*	−0.124	0.204	1	**0.619**	0.082	**-0.310**
	*p*	0.401	0.164		**0.000**	0.581	**0.032**

OSI stable period	*R*	0.071	**−0.543**	**0.619**	1	−0.028	−0.099
	*p*	0.633	**0.000**	**0.000**		0.850	0.502

VEGF stable period	*R*	0.265	0.191	0.082	−0.028	1	−0.046
	*p*	0.068	0.193	0.581	0.850		0.758

TNF-*α* stable period	*R*	0.034	−0.206	**−0.310**	−0.099	−0.046	1
	*p*	0.817	0.160	**0.032**	0.502	0.758	

*Note:* Values corresponding to the significance level *p* < 0.05 are marked in bold. The *p* value was obtained from the Spearman test.

Abbreviations: OSI = oxidative stress index, TAS = total antioxidant status, TNF-*α* = tumor necrosis factor-alpha, TOS = total oxidant status, VEGF = vascular endothelial growth factor.

**Table 6:** Generalized linear regression analysis of factors affecting kallistatin levels.

Variable	Multivariate generalized linear model	
	*β* coefficient (95% CI)	*p*
Age	1.586 (−0.421–3.594)	0.121
Sex (ref = female)	−50.926 (−120.439–18.587)	0.151
Pneumonia (ref = no)	−0.546 (−40.784–39.691)	0.949
OSI exacerbation period (AU)	16.889 (2.203–31.574)	**0.049**
VEGF exacerbation period (pg/mL)	1.191 (−0.049–2.432)^∗^	0.060

*Note:* Values corresponding to the significance level *p* < 0.05 are marked in bold.

Abbreviations: OSI = oxidative stress index, VEGF = vascular endothelial growth factor.

^∗^Due to the nonparametric distribution of VEGF, its logarithmic value was used for calculations.

## Data Availability

The data supporting the findings of this study are available from the corresponding author upon reasonable request.
